# Creatine homeostasis and the kidney: comparison between kidney transplant recipients and healthy controls

**DOI:** 10.1007/s00726-024-03401-w

**Published:** 2024-06-13

**Authors:** Adrian Post, Dion Groothof, Daan Kremer, Tim J. Knobbe, Willem Abma, Christa A. Koops, Dimitrios Tsikas, Theo Wallimann, Robin P.F. Dullaart, Casper F.M. Franssen, Ido P. Kema, M. Rebecca Heiner-Fokkema, Stephan J.L. Bakker

**Affiliations:** 1 Department of Internal Medicine, University Medical Center Groningen, University of Groningen, Groningen, 9713 GZ The Netherlands; 2 Department of Laboratory Medicine, University of Groningen, University Medical Center Groningen, Groningen, 9713 GZ the Netherlands; 3 Institute of Toxicology, Core Unit Proteomics, Hannover Medical School, Carl-Neuberg-Str. 1, 30625 Hannover, Germany; 4 Department of Biology, ETH Zurich, Zurich, Switzerland; 5 Department of Internal Medicine, Division of Nephrology, University Medical Center Groningen, Groningen, 9700 RB the Netherlands

**Keywords:** Creatine, Guanidinoacetate, Kidney transplant recipients, Measured glomerular filtration rate, Epidemiology

## Abstract

Creatine is a natural nitrogenous organic acid that is integral to energy metabolism and crucial for proper cell functioning. The kidneys are involved in the first step of creatine production. With kidney transplantation being the gold-standard treatment for end-stage kidney disease, kidney transplant recipients (KTR) may be at risk of impaired creatine synthesis. We aimed to compare creatine homeostasis between KTR and controls. Plasma and urine concentrations of arginine, glycine, guanidinoacetate, creatine and creatinine were measured in 553 KTR and 168 healthy controls. Creatine intake was assessed using food frequency questionnaires. Iothalamate-measured GFR data were available in subsets of 157 KTR and 167 controls. KTR and controls had comparable body weight, height and creatine intake (all *P* > 0.05). However, the total creatine pool was 14% lower in KTR as compared to controls (651 ± 178 vs. 753 ± 239 mmol, *P* < 0.001). The endogenous creatine synthesis rate was 22% lower in KTR as compared to controls (7.8 ± 3.0 vs. 10.0 ± 4.1 mmol per day, *P* < 0.001). Despite lower GFR, the plasma guanidinoacetate and creatine concentrations were 21% and 41% lower in KTR as compared to controls (both *P* < 0.001). Urinary excretion of guanidinoacetate and creatine were 66% and 59% lower in KTR as compared to controls (both *P* < 0.001). In KTR, but not in controls, a higher measured GFR was associated with a higher endogenous creatine synthesis rate (std. beta: 0.21, 95% CI: 0.08; 0.33; *P* = 0.002), as well as a higher total creatine pool (std. beta: 0.22, 95% CI: 0.11; 0.33; *P* < 0.001). These associations were fully mediated (93% and 95%; *P* < 0.001) by urinary guanidinoacetate excretion which is consistent with production of the creatine precursor guanidinoacetate as rate-limiting factor. Our findings highlight that KTR have a disturbed creatine homeostasis as compared to controls. Given the direct relationship of measured GFR with endogenous creatine synthesis rate and the total creatine pool, creatine supplementation might be beneficial in KTR with low kidney function.

Trial registration ID: NCT02811835.

**Trial registration URL**: https://clinicaltrials.gov/ct2/show/NCT02811835.

**Supplementary Information:**

The online version contains supplementary material available at 10.1007/s00726-024-03401-w.

## Introduction

Creatine is a natural nitrogenous organic acid that is integral to energy metabolism and crucial for proper cell functioning (Wallimann and Harris 2016). The main role of creatine is temporal and spatial energy buffering in the creatine kinase/phosphocreatine system. It has been estimated that adult men weighing 70 kg have approximately 120 g of creatine (creatine and creatine phosphate combined); however, this quantity varies markedly with muscle mass (Brosnan et al. 2011), where over 95% of the total creatine pool resides. Various studies on creatine metabolism demonstrated that creatine and phosphocreatine are both non-enzymatically converted to creatinine at a constant rate of about 1.7% of the total creatine pool per day (Borsook and Dubnoff 1947; Crim et al. 1975; Kan et al. 2006). The resulting creatinine diffuses out of the cell and is excreted unchanged in urine through glomerular filtration and to a lesser extent by tubular secretion. Under steady state conditions, urinary creatinine excretion is therefore a good marker of a person’s total body creatine pool. Since most of the creatine pool resides in skeletal muscles, the total creatine pool, as measured by the 24-h urinary creatinine excretion, is also considered the gold-standard biochemical marker of muscle mass (Heymsfield et al. 1983; Oterdoom et al. 2009; McCarthy et al. 2022; van Vliet et al. 2022).

Importantly, because of the constant loss of creatine by conversion to creatinine, there is a need for a continuous supply of creatine to remain in steady-state and maintain muscle mass homeostasis. In omnivores, this supply is a combination of dietary creatine intake and endogenous creatine synthesis. Dietary sources of creatine are primarily animal-based foods (meat, poultry, fish, and seafood) and dairy products (Bakian et al. 2020; Korovljev et al. 2021; Todorovic et al. 2022). Endogenous creatine synthesis consists of two steps. The first and rate-limiting step of creatine synthesis is the transfer of the amidino group of arginine to glycine, catalyzed by the enzyme L-arginine: glycine amidinotransferase (AGAT), yielding guanidinoacetate (Walker 1979; Wyss and Kaddurah-Daouk 2000). The second step is the methylation of guanidinoacetate, catalyzed by the enzyme guanidinoacetate N-methyltransferase (GAMT), yielding creatine. Early non-clinical studies demonstrated that livers from rats, mice, dogs, cats, and rabbits lack AGAT activity. On the basis of these findings and on the fact that the rate of creatine biosynthesis is considerably reduced in nephrectomized animals (Goldman and Moss 1960; Fitch et al. 1961; Levillain et al. 1995), it has become widely accepted that creatine biosynthesis in mammals involves production of guandinoacetate in the kidneys and subsequent methylation in the liver, after which creatine is transported and taken up by various tissues (Wyss and Kaddurah-Daouk 2000). It should be noted, however, that this line of thought has been debated, with studies demonstrating that AGAT activity is also present in high amounts in the pancreas and in lower amounts in the brain, liver and spleen of humans, although when accounted for organ weight all in lower amounts than in the kidneys (Van Pilsum et al. 1972). The exact contribution of the kidney to creatine synthesis remains to be determined, because clinical data on creatine synthesis in patients with kidney impairment are scarce or even absent. Nonetheless, it is plausible to hypothesize that if guanidinoacetate production is already a rate limiting step under physiological circumstances, this becomes even more pronounced as kidney function declines.

With kidney transplantation being the gold-standard treatment for end-stage kidney disease, kidney transplant recipients (KTR) are an increasingly prevalent group of patients (Lentine et al. 2023). Clearly, this group may be at risk of impaired creatine synthesis. While post-transplant care is increasingly improved, it remains unknown how KTR compare to controls with regard to alterations in many physiological processes, including creatine homeostasis. The importance of proper creatine homeostasis has been described in the KTR population, with previous findings demonstrating that KTR with lower creatine pools - as reflected by lower 24-h urinary creatinine excretion - are more prone to premature mortality, graft failure, impaired mental and physical health-related quality of life (Oterdoom et al. 2008; van Vliet et al. 2022; Post et al. 2023), whereas lower plasma creatine concentrations and estimated intramuscular creatine concentrations were associated with a higher prevalence of self-reported fatigue (Post et al. 2023). However, to date, no studies have investigated how KTR differ from controls with regard to creatine intake, creatine pools, endogenous creatine synthesis and biomarkers of creatine synthesis. Furthermore, the relationship of measures of kidney function with creatine homeostasis is also unknown. Therefore, in the current study we aimed to:

1) compare creatine intake, the total creatine pool, and the endogenous creatine synthesis rate between KTR and controls,

2) compare the plasma concentrations, urinary excretions and fractional excretions of creatine, and the major creatine-related biomarkers: arginine, glycine, guanidinoacetate between KTR and controls,

3) investigate the associations of the endogenous creatine synthesis rate and the total creatine pool with creatine-related biomarkers, demographics, kidney and liver function markers, and clinical characteristics in KTR and controls.

4) investigate associations of iothalamate-measured glomerular filtration rate (measured GFR) with the aforementioned creatine homeostasis markers and, where appropriate, investigate whether such associations are mediated by urinary guanidinoacetate excretion (as a potential marker of the production of the precursor of creatine).

## Methods

### Study population

This observational prospective study was conducted in the TransplantLines Food and Nutrition Biobank and Cohort Study, a cohort of KTR that has been described in detail previously (van den Berg et al. 2012; Berg et al. 2014; Post et al. 2021a). In short, all adult (≥ 18 years old) prevalent KTR without known or apparent systemic illnesses (i.e., malignancies, opportunistic infections) who visited the outpatient clinic of the University Medical Center Groningen between November 2008 and June 2011 were invited to participate. Geographically, the cohort is based on KTR living in the northern part of the Netherlands. Included KTR were all transplanted at the University Medical Center Groningen and had no history of drug or alcohol addiction. Of 817 initially invited KTR, 706 (87%) signed written informed consent to participate in the study. After excluding KTR with missing data on endogenous creatine synthesis rate or biomarkers of creatine synthesis (urine and plasma concentrations of arginine, glycine, guanidinoacetate or creatine), a total of 553 KTR were eligible for the majority of statistical analyses. As a control group, healthy potential kidney donors in screening for kidney donation were used. A total of 300 potential donors were invited, of whom 297 signed informed consent and of which 196 actually donated a kidney. Exclusion criteria for the healthy controls were insufficient understanding of the Dutch language, a history of alcohol or drug abuse, diabetes mellitus, disease that would make them unsuitable for donation, a medical history of cardiovascular events, and usage of more than two antihypertensives. After excluding donors with missing data on endogenous creatine synthesis rate or biomarkers of creatine synthesis (urine and plasma concentrations of arginine, glycine, guanidinoacetate or creatine), a total of 168 donors were eligible for statistical analyses. One donor did not have data on measured GFR. Iothalamate-measured GFR data were available in subsets of 157 KTR and 167 controls.

A flowchart of KTR and donors through the study is shown in Figure S1. The study protocol was approved by the University Medical Center Groningen institutional ethical review board (Medical ethical committee 2008/186) and was conducted in accordance with the Declaration of Helsinki and Declaration of Istanbul. The reporting of the current study conforms to the EQUATOR guideline: The Strengthening the Reporting of Observational Studies in Epidemiology (von Elm et al. 2007).

### Laboratory measurements

All participants were instructed to collect a 24-hour urine sample according to a strict protocol on the day before their visit to the outpatient clinic. Urine was collected under oil and chlorhexidine that was added as an antiseptic agent. Fasting venous blood samples treated with lithium-heparin, sodium-fluoride or potassium-EDTA were obtained in the morning. Samples were divided in small aliquots and immediately stored at − 80 ◦C for later use. Concentrations of arginine, glycine, creatine and creatinine were determined in plasma and urine with a hydrophilic interaction liquid chromatography (HILIC) method coupled to tandem mass spectrometry. In short: To 50 µL urine or plasma, 20 µL internal standard mix was added containing the stable isotopes D_3−_creatine, D_3_-creatinine, ^13^C_2_^15^N -glycine and ^13^C_2_-guanidinoacetate (only for urine samples). This was followed by 150 µL of acetonitrile mixed with 0.1% formic acid for protein precipitation. Samples were then mixed and centrifuged. One µL of sample volume was injected into the LC-MS/MS system. Chromatographic separation was achieved during a 14-minute run using an ACQUITY UPLC BEH Amide Column (130 Å, 1.7 μm, 2.1 mm × 100 mm) (Waters, Milford, MA, USA) kept at 40 °C and a Nexera UHPLC system (Shimadzu, Kioto, Japan). Mobile phase A consisted of 0.1 v/v% formic acid and 10 mM ammonium formate in Milli-Q water and mobile phase B of 0.1 v/v% formic acid and 10 mM ammonium formate in 95 v/v% acetonitrile/Milli-Q water. Total flow was set at 0.4 mL/min and the gradient elution was as follows: 0 min 90% B, 1.5 min 90%B, 6.0 min 75% B, 8.0 min 72% B, 8.1 min 50% B, 10 min 50% B, 10.1 min 90% B, 14.0 min 90% B. A Sciex 4500 QTrap mass spectrometer (Sciex, Framingham, MA, USA) was used in positive electrospray ionization mode, using nitrogen as collision, carrier, and curtain gas. Sciex Analyst®MD 1.6.3 and Sciex Multiquant®MD 3.0.3 (Sciex, Framingham, MA, USA) were used for data acquisition and processing. Plasma guanidinoacetate concentration were previously determined using a gas chromatograph-single quadrupole mass spectrometer (ThermoFisher, Dreieich, Germany) (Hanff et al. 2019).

ALAT, ASAT, bilirubin, total protein, hs-CRP, glucose, hemoglobin, glucose, homocysteine, vitamin B12, folic acid and urinary urea excretion were measured using routine clinical laboratory methods (Roche Diagnostics, Basel, Switzerland). Plasma vitamin B6 was measured as pyridoxal-5-phospate by means of a validated HPLC method (Waters Alliance) with fluorescence detection (FP-2020; Jasco Inc.) Serum cystatin C was measured with the Gentian Cystatin C Immunoassay (Gentian AS, Moss, Norway) on a Roche Modular analyzer and was calibrated directly with the standard supplied by the manufacturer. The measured GFR was assessed using low-dose ^125^I-iothalamate infusion as previously described (Apperloo et al. 1996). The day-to-day measured GFR variability was found to be 2.5%.

### Clinical parameters and dietary intake

Body weight and height were measured wearing indoor clothing without shoes. Physical activity was assessed using the Short Questionnaire to Assess Health-Enhancing Physical Activity (SQUASH) score in time multiplied by intensity.

Information on dietary intake was obtained from a validated semiquantitative food-frequency questionnaire (FFQ), which was linked to the Dutch food composition table (NEVO) (Feunekes et al. 1993). The FFQ inquired about intakes of 177 food items during the past month with seasonal variations taken into account. For each item, the frequency was recorded in times per day, week, or month. The number of servings was expressed in natural units (e.g., slice of bread or apple) or household measures (e.g., cup or spoon). The questionnaire was self-administered and filled out at home. Every FFQ was checked for completeness by a trained researcher and inconsistent answers were verified with the patients. Validation of the FFQ was assessed as previously reported (van den Berg et al. 2013). Dietary data were converted into daily nutrient intakes with the use of the Dutch Food Composition Table of 2006 (for Public Health and the Environment). The daily intake of meat, fish and dairy was used to calculate the daily creatine intake. Individual values for total grams of creatine consumed per day were computed using the average amount of creatine (0.20 g/kg for dairy-based foods and 3.88 g/kg for meat and fish) across all creatine-containing food sources, as described in (Bakian et al. 2020; Korovljev et al. 2021; Todorovic et al. 2022).

### Definitions

The total creatine pool was defined as the 24-h urinary excretion of creatinine divided by 0.017, given the daily degradation of 1.7% of the creatine pool into creatinine, which is excreted into the urine (Crim et al. 1975; Walker 1979). The endogenous creatine synthesis rate was defined as the combined 24-h urinary excretion of creatine plus creatinine, minus the daily dietary intake of creatine (Brosnan et al. 2011).

Fractional excretions of arginine, glycine, guanidinoacetate and creatine were calculated as the clearance of the arginine, glycine, guanidinoacetate and creatine, divided by the clearance of creatinine, times 100%. High fractional excretions imply low rates of reabsorption, and fractional excretions larger than 100% imply net tubular secretion, rather than reabsorption. Clearance is calculated as the urinary excretion from a 24-hour urine collection divided by the plasma concentration and expressed in ml/min. As a sensitivity analysis, we also included fractional excretions calculated using measured GFR, rather than creatinine clearance, in a subset of patients with data on both measured GFR and creatinine clearance.

### Statistical analyses

All data analyses and computations were performed with R software, version 4.3.0 (The R-Foundation for Statistical Computing). Baseline data are presented as mean ± standard deviation for normally distributed data, median [interquartile range] for non-normally distributed data, and number (percentage) for nominal data. Data on creatine pools, creatine intake, endogenous creatine synthesis rate, as well as data on plasma concentrations, urinary excretions and fractional excretions of arginine, glycine, guanidinoacetate and creatine are presented both for the entire study population as well as stratified based on sex, given the previously identified sex-based differences in circulating creatine concentrations and creatinine excretion (Post et al. 2020). Overlapping dot and box-plots are used to present the distribution of creatine pool, creatine intake and endogenous creatine synthesis rate, using the quasirandom geom function in R to offset points within categories to reduce overplotting. Differences in baseline characteristics and creatine-related variables between KTR and controls were tested using the Student t-test, Wilcoxon signed-rank test and Chi-squared test, where appropriate. In the main cohort, no data on muscle mass were available. To assess whether the differences in creatine pools between KTR and controls could be driven by differences in muscle mass, we included a sensitivity analysis with data from another KTR and control cohort, as described previously (Eisenga et al. 2018). In this sensitivity analysis, muscle mass was estimated via bioelectrical impedance analysis according to the equations developed by Sergi et al. (Sergi et al. 2015), Kyle et al. (Kyle et al. 2004) and Janssen et al. (Janssen et al. 2000).

Associations of the endogenous creatine synthesis rate and the total creatine pool with plasma concentrations, urinary excretions, and fractional excretions of arginine, glycine, guanidinoacetate and creatine, as well as demographic, kidney, liver and other variables, were studied using linear regression analyses. Similarly, for the second part of the study, associations of the measured GFR with endogenous creatine synthesis rate, total creatine pool, creatine intake and plasma concentrations, urinary excretions, and fractional excretions of arginine, glycine, guanidinoacetate and creatine, were also studied using linear regression analyses. Where applicable, logarithmic, square root or cubic root transformation were applied to ensure that the model assumptions for linear regression analyses were met. All analyses were adjusted for the *a priori* defined potential confounders age, sex, body weight, height, and time since transplantation (for KTR). Coefficients were presented as standardized betas, referring to the number of standard deviations a dependent variable changes per standard deviation increase of the independent variable. The number of variables with missing data was smaller than 1%, therefore no multiple imputations were used to account for missing data.

To assess whether the association of measured GFR with endogenous creatine synthesis rate and total creatine pool was mediated by the urinary guanidinoacetate excretion (as a potential marker of AGAT activity), mediation analyses were conducted using the mediation package in R. A total of 1000 bootstrap simulations were run to generate estimates and confidence intervals for mediation effects. Mediation analyses were also adjusted for the *a priori* defined potential confounders age, sex, body weight, height and time since transplantation (for KTR). The proportion mediated, is defined as the indirect path divided by the total path times 100%, and quantifies the extent to which the influence of the independent variable on the outcome variable (dependent variable) is conveyed through the mediator variable. It indicates the fraction of the total effect of the treatment variable on the outcome variable that is attributable to the mediator variable. A value of 0% suggests that the mediator has no impact on the relationship between the treatment variable and the outcome variable, indicating no mediation. Conversely, a value of 100% implies that the entire effect of the treatment variable on the outcome variable is mediated through the mediator, suggesting complete mediation. The visreg package was used to visualize the adjusted linear regression analyses of the mediation analyses.

## Results

A total of 553 KTR and 168 controls were included for the first part of the statistical analyses. A CONSORT flowchart of KTR and controls throughout the study is shown in Figure S1. KTR and controls were comparable in age (53 ± 13 vs. 54 ± 11 years), weight (80 ± 16 vs. 80 ± 14 kg), and height (174 ± 10 vs. 175 ± 9 cm). KTR were more often male (57% vs. 47%) and had lower eGFR_creat & cys C_ (47 ± 20 vs. 98 ± 15 mmol/L). A full tabulated overview of all baseline characteristics is shown in Table S1.

### Creatine pool

**Fig. 1:**
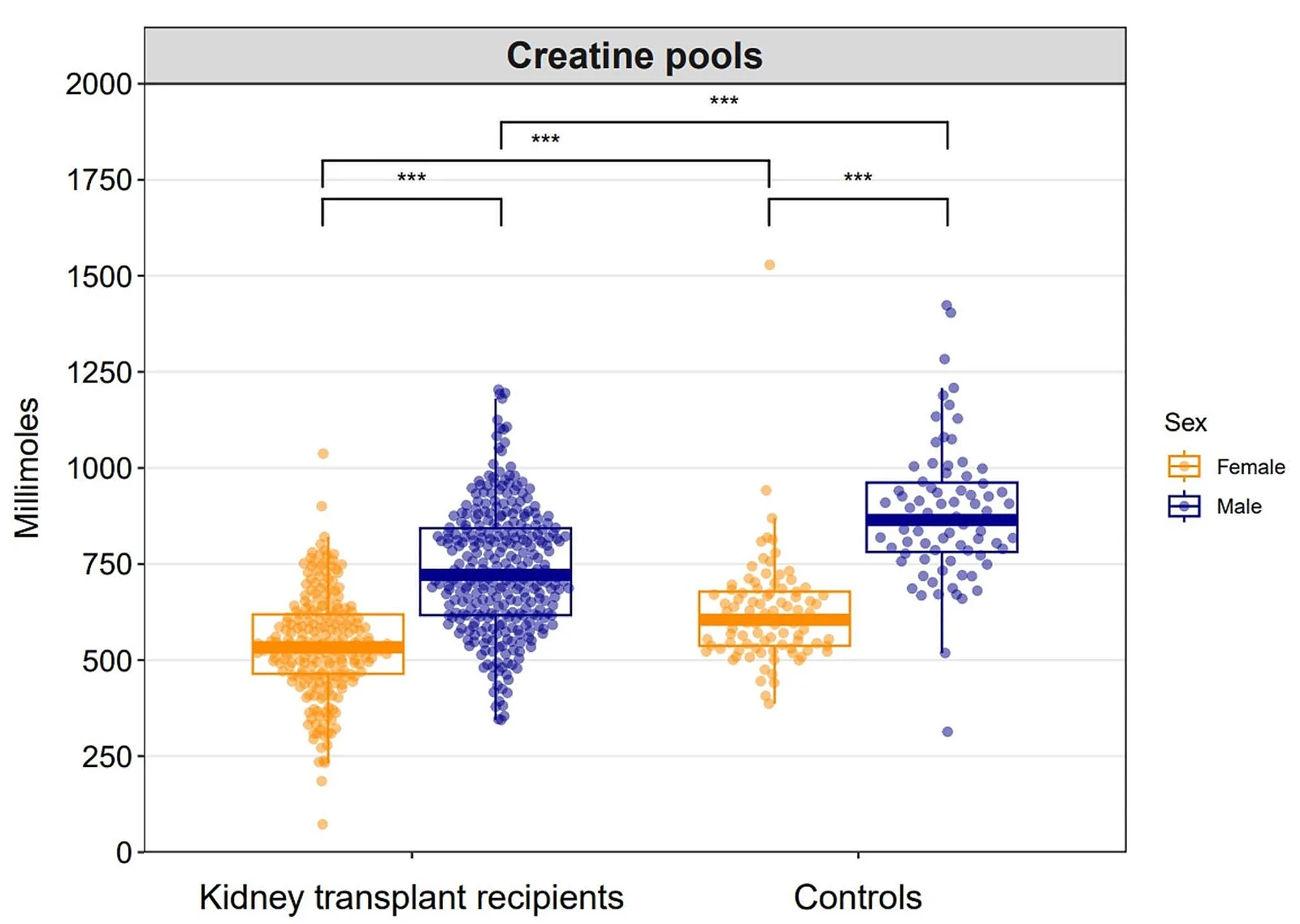
Combined dot plot and box plot demonstrating the distributions of creatine pools in kidney transplant recipients and controls according to sex. The quasirandom geom function in R was used to offset points within categories to reduce overplotting

The total creatine pool was 651 ± 178 mmol in KTR and 753 ± 239 mmol in controls (*P* < 0.001), indicating that the total creatine pool was 14% lower in KTR as compared to controls. In KTR, the total creatine pool was 539 ± 133 mmol for females and 734 ± 162 mmol for males (*P* < 0.001). In controls, the total creatine pool was 623 ± 141 mmol for females and 900 ± 243 mmol for males (*P* < 0.001). A graphical overview of total creatine pools for KTR and controls according to sex, is shown in Fig. 1. Sensitivity analyses performed in a comparable cohort suggest that the differences in creatine pools are not explained by differences in muscle mass, Table S2.

### Dietary creatine intake

**Fig. 2:**
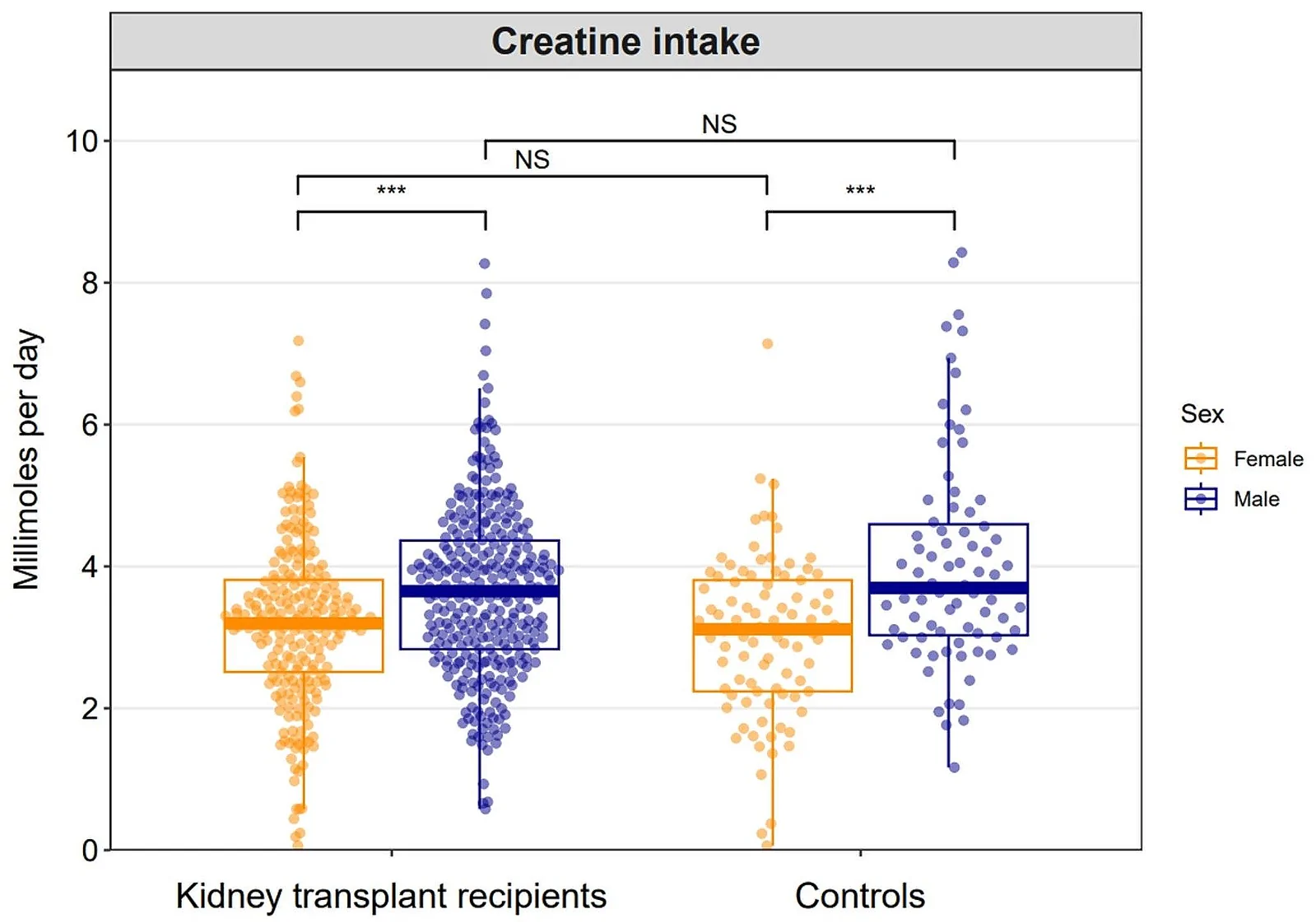
Combined dot plot and box plot demonstrating the distributions of dietary creatine intake in kidney transplant recipients and controls according to sex. The quasirandom geom function in R was used to offset points within categories to reduce overplotting

The dietary creatine intake was estimated to be 3.5 ± 1.2 mmol per day in KTR and 3.5 ± 1.4 mmol per day in controls (P for difference = 0.5). In KTR, the dietary creatine intake was estimated to be 3.2 ± 1.2 mmol per day in females and 3.7 ± 1.2 mmol per day in males (P for difference < 0.001). In controls, the dietary creatine intake was estimated to be 3.0 ± 1.1 mmol per day for females and 4.0 ± 1.5 mmol per day for males (P for difference < 0.001). A graphical overview of dietary creatine intake for KTR and controls according to sex, is shown in Fig. 2.

### Endogenous creatine synthesis rate

**Fig. 3:**
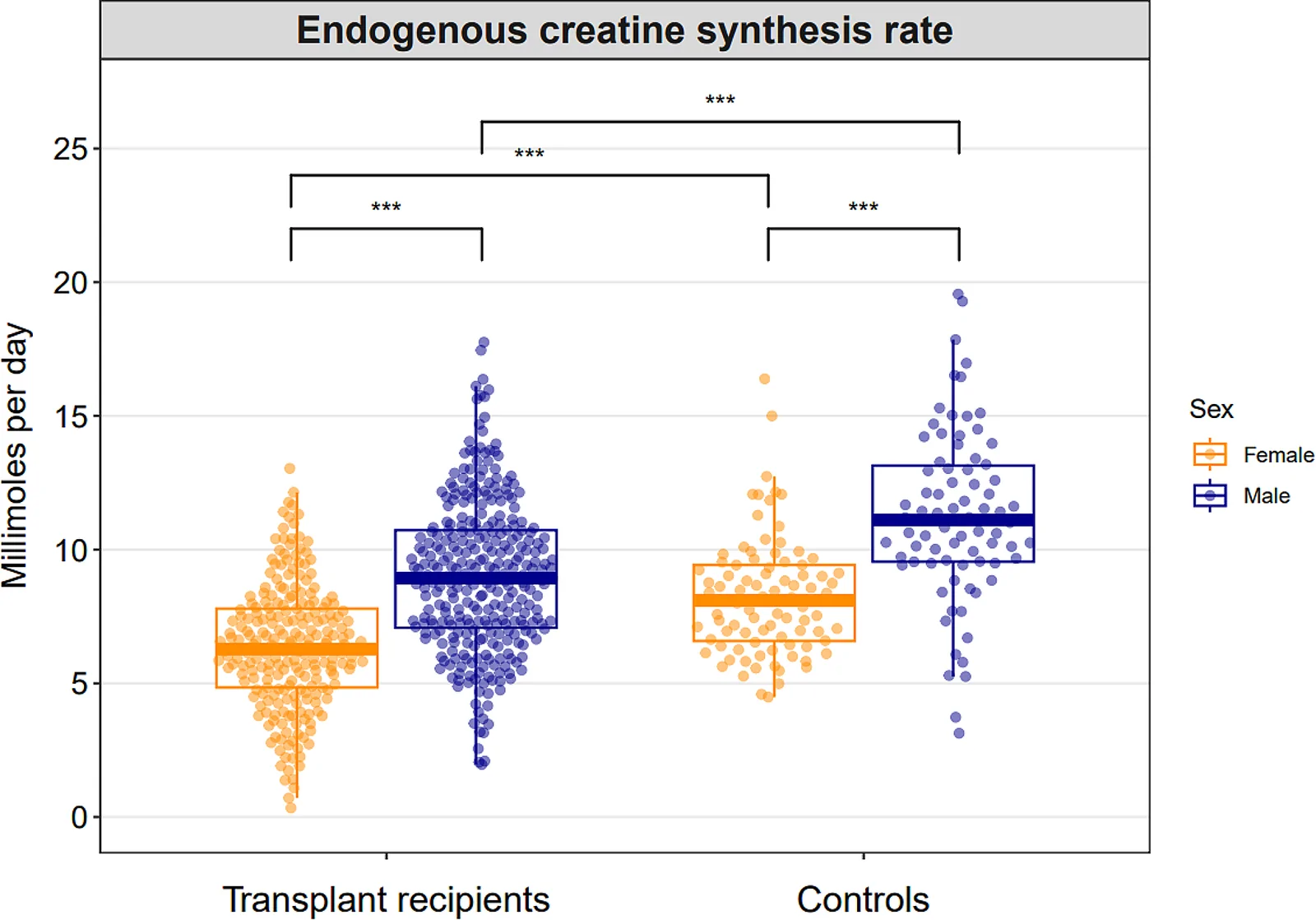
Combined dot plot and box plot demonstrating the distributions of endogenous creatine synthesis rate in kidney transplant recipients and controls according to sex. The quasirandom geom function in R was used to offset points within categories to reduce overplotting

The endogenous creatine synthesis rate was 7.8 ± 3.0 mmol per day in KTR and 10.0 ± 4.1 mmol per day in controls (P for difference < 0.001), indicating that the total creatine pool was 22% lower in KTR as compared to controls. In KTR, the endogenous creatine synthesis rate was 6.3 ± 2.4 mmol per day for females and 9.0 ± 2.9 mmol per day for males (P for difference < 0.001). In controls, the endogenous creatine synthesis rate was 8.5 ± 3.3 mmol for females and 11.6 ± 4.4 mmol for males (P for difference < 0.001). A graphical overview of the endogenous creatine synthesis requirement for KTR and controls according to sex, is shown in Fig. 3.

### Plasma concentrations, urinary excretions and fractional excretions of arginine, glycine, guanidinoacetate and creatine

Plasma concentrations of arginine (92 ± 31 vs. 73 ± 25 µmol/L) were higher in KTR as compared to controls, whereas plasma concentrations of guanidinoacetate (2.2 [1.8; 2.7] vs. 2.8 [2.4; 3.4] µmol/L) and creatine (22 [15 ; 37] vs. 37 [25; 49] µmol/L) were lower in KTR as compared to controls. Plasma creatine concentrations were higher in females as compared to males, in both KTR (31 [19; 48] vs. 19 [13; 28] µmol/L) and controls (43 [33; 55] vs. 27 [21; 41] µmol/L). Sex-based differences were less evident for arginine, glycine and guanidinoacetate.

Urinary excretions of arginine (22 [16; 33] vs. 22 [17; 31] µmol/24 h) were comparable between KTR and controls, whereas the urinary excretion of glycine (0.7 [0.4; 1.3] vs. 1.7 [1.1; 2.4] mmol/24 h), guanidinoacetate (139 [72–232] vs. 405 [308–525] µmol/24 h) and creatine (98 [62; 168] vs. 238 [123; 639] µmol/24 h) were lower in KTR as compared to controls. Urinary creatine excretion was higher in females as compared to males, but only in controls (328 [117; 1129] vs. 177 [129; 334] µmol/24 h). Sex-based differences were less evident for arginine, glycine and guanidinoacetate.

The fractional excretion of arginine was < 1% and the fractional excretion of glycine was roughly 4% in both KTR and controls, in line with extensive reabsorption. The fractional excretion of guanidinoacetate was high and nearing 100% in both KTR and controls, indicative of little limited net reabsorption. The fractional excretion of creatine (6.2 [3.5; 11.4] vs. 4.0 [2.4; 8.7] %) was higher in KTR as compared to controls, which was driven by an effect in males. A full tabulated comparison of plasma concentrations, urinary excretions and fractional excretion of arginine, glycine, guanidinoacetate and creatine in KTR and controls is shown in Table 1. A sensitivity analysis demonstrated that the results of the fractional excretions are similar when calculated using creatinine clearance or measured GFR, as shown in Table S3.

**Table 1:** Creatine and its precursors in KTR and controls

		KTR (*n* = 553)	Controls (*n* = 168)	*P*-value
**Plasma concentrations**				
Arginine, µmol/L				
	All	92 ± 31	73 ± 25	< 0.001
	Females	88 ± 28	73 ± 27	< 0.001
	Males	95 ± 33	74 ± 22	< 0.001
Glycine, µmol/L				
	All	229 ± 69	222 ± 75	0.2
	Females	238 ± 76	239 ± 88	0.9
	Males	223 ± 64	200 ± 47	< 0.001
Guanidinoacetate, µmol/L				
	All	2.2 [1.8; 2.7]	2.8 [2.4; 3.4]	< 0.001
	Females	2.1 [1.7; 2.6]	2.7 [2.3; 3.4]	< 0.001
	Males	2.2 [1.8; 2.8]	2.9 [2.7; 3.5]	< 0.001
Creatine, µmol/L				
	All	22 [15 ; 37]	37 [25; 49]	< 0.001
	Females	31 [19; 48]	43 [33; 55]	< 0.001
	Males	19 [13; 28]	27 [21; 41]	< 0.001
**Urinary excretion**				
Arginine, µmol/24 h				
	All	22 [16; 33]	22 [17; 31]	0.9
	Females	21 [15; 30]	21 [16; 29]	0.5
	Males	24 [16; 36]	24 [17; 33]	0.9
Glycine, mmol/24 h				
	All	0.7 [0.4; 1.3]	1.7 [1.1; 2.4]	< 0.001
	Females	0.7 [0.4; 1.2]	1.6 [1.0; 2.2]	< 0.001
	Males	0.8 [0.5; 1.3]	1.9 [1.3; 2.5]	< 0.001
Guanidinoacetate, µmol/24 h				
	All	139 [72; 232]	405 [308; 525]	< 0.001
	Females	143 [63; 235]	404 [327; 515]	< 0.001
	Males	136 [74; 229]	410 [298; 546]	< 0.001
Creatine, µmol/24 h				
	All	98 [64; 170]	239 [123; 613]	< 0.001
	Females	94 [51; 344]	321 [118; 1089]	< 0.001
	Males	99 [73; 141]	173 [128; 322]	< 0.001
**Fractional renal excretion**				
Arginine, %				
	All	0.30 [0.20; 0.49]	0.18 [0.31; 0.24]	< 0.001
	Females	0.33 [0.22; 0.52]	0.20 [0.14; 0.25]	< 0.001
	Males	0.28 [0.19; 0.49]	0.16 [0.12; 0.22]	< 0.001
Glycine, %				
	All	3.9 [2.5; 6.9]	4.5 [3.0; 5.9]	0.2
	Females	4.1 [2.7; 7.4]	4.4 [2.8; 5.9]	0.8
	Males	3.7 [2.3; 6.8]	4.7 [3.2; 6.2]	0.03
Guanidinoacetate, %				
	All	71 [43; 112]	80 [55; 112]	0.2
	Females	88 [47; 130]	97 [67; 140]	0.3
	Males	65 [42; 99]	70 [50; 86]	0.8
Creatine, %				
	All	6.2 [3.5; 11.4]	4.0 [2.4; 8.7]	< 0.001
	Females	5.0 [2.6; 11.8]	5.5 [3.0; 13.6]	0.5
	Males	6.4 [3.8; 10.2]	3.3 [2.4; 4.8]	< 0.001

### Associations of endogenous creatine synthesis rate with creatine-related clinical and laboratory parameters

Both in KTR and controls, a higher total creatine pool was associated with a higher endogenous creatine synthesis rate, whereas a higher dietary creatine intake was associated with a lower endogenous creatine synthesis rate. In KTR, but not controls, both higher plasma guanidinoacetate concentration and higher plasma creatine concentration were associated with a higher endogenous creatine synthesis rate. In both KTR and controls, both a higher urinary guanidinoacetate excretion and a higher urinary creatine excretion were associated with a higher endogenous creatine synthesis rate. In KTR, a higher fractional guanidinoacetate excretion was associated with a higher endogenous creatine synthesis rate. A full overview of the linear regression analyses with endogenous creatine synthesis rate as dependent variable is shown in Table 2. Comparable results were found when the total creatine pool was used as the dependent variable, rather than endogenous creatine synthesis. A full overview of the linear regression analyses of total creatine pool with various creatine parameters is shown in Table S4.

**Table 2:** Associations of creatine parameters with the endogenous creatine synthesis rate in KTR and controls

	KTR (*n* = 553)		Controls (*n* = 168)	
Dependent variable:	Std. Beta (95% CI)	*P*-value	Std. Beta (95% CI)	*P*-value
**Creatine status**				
Total creatine pool	0.96 (0.92; 1.02)	< 0.001	1.05 (0.96; 1.13)	< 0.001
Creatine intake	-0.27 (-0.34; -0.21)	< 0.001	-0.25 (-0.39; -0.11)	< 0.001
**Plasma concentrations**				
Arginine	-0.01 (-0.08; 0.06)	0.76	-0.01 (-0.14; 0.13)	0.96
Glycine	0.02 (-0.05; 0.09)	0.58	0.02 (-0.05; 0.09)	0.55
Guanidinoacetate	0.07 (0.01; 0.14)	0.04	0.02 (-0.10; 0.13)	0.80
Creatine	0.12 (0.05; 0.19)	0.002	0.08 (-0.07; 0.23)	0.32
**Urinary excretions**				
Arginine	0.15 (0.09; 0.22)	< 0.001	0.26 (0.13; 0.39)	< 0.001
Glycine	0.21 (0.14; 0.28)	< 0.001	0.11 (-0.03; 0.25)	0.11
Guanidinoacetate	0.31 (0.25; 0.37)	< 0.001	0.33 (0.20; 0.45)	< 0.001
Creatine	0.27 (0.20; 0.33)	< 0.001	0.27 (0.13; 0.40)	< 0.001
**Fractional excretion**				
Arginine	-0.08 (-0.14; -0.01)	0.025	0.03 (-0.09; 0.14)	0.64
Glycine	-0.01 (-0.07; 0.06)	0.90	-0.06 (-0.17; 0.05)	0.27
Guanidinoacetate	0.15 (0.08; 0.21)	< 0.001	0.01 (-0.12; 0.13)	0.94
Creatine	0.06 (-0.01; 0.13)	0.07	0.16 (0.05; 0.27)	0.004

Analyses are adjusted for age, sex, weight, height and time since transplantation (for KTR)

In KTR, higher weight, height, kidney function, hemoglobin, physical activity and protein intake were all associated with a higher endogenous creatine synthesis. Higher vitamin B6 concentration, higher sulfate excretion and higher taurine excretion were also associated with higher endogenous creatine synthesis. Higher age, higher homocysteine, higher hs-CRP, higher NT-proBNP and higher glucose were all associated with lower endogenous creatine synthesis. In controls, many of these associations had a similar direction, but did not reach statistical significance. ALAT, ASAT, bilirubin and total protein concentration were not associated with endogenous creatine synthesis rate in either KTR or controls. A full overview of the linear regression analyses of endogenous creatine synthesis rate with demographics, kidney and liver function markers, transsulfuration-related markers and clinical characteristics is shown in Table 3. Comparable results were found when total creatine pool was used as the dependent variable, rather than endogenous creatine synthesis. A full overview of the linear regression analyses of total creatine pool with demographics, kidney and liver function markers, transsulfuration related markers and clinical characteristics is shown in Table S5.

**Table 3:** Associations of clinical and biochemical parameters with the endogenous creatine synthesis rate in KTR and controls

	KTR (*n* = 553)		Controls (*n* = 168)	
Dependent variables:	Std. Beta (95% CI)	*P*-value	Std. Beta (95% CI)	*P*-value
**Demographics**				
Age	-0.21 (-0.28; -0.14)	< 0.001	-0.22 (-0.35; -0.08)	< 0.001
Sex, male	0.47 (0.28; 0.65)	< 0.001	0.37 (0.01; 0.74)	0.045
Weight	0.29 (0.21; 0.37)	< 0.001	0.47 (0.29; 0.64)	< 0.001
Height	0.17 (0.07; 0.27)	< 0.001	-0.10 (-0.30; 0.12)	0.40
**Kidney related**				
eGFR Creat + Cys C 2021	0.12 (0.05; 0.19)	< 0.001	-0.04 (-0.19; 0.12)	0.67
eGFR Creat 2021	0.04 (-0.03; 0.11)	0.22	-0.08 (-0.24; 0.08)	0.34
eGFR Cys C 2012	0.16 (0.09; 0.22)	< 0.001	-0.01 (-0.16; 0.15)	0.98
Creatinine clearance	0.32 (0.26; 0.39)	< 0.001	0.16 (0.05; 0.28)	0.005
**Liver related**				
ALAT	0.03 (-0.04; 0.10)	0.44	0.02 (-0.12; 0.16)	0.74
ASAT	0.03 (-0.04; 0.09)	0.40	0.08 (-0.05; 0.22)	0.25
Bilirubin	0.02 (-0.05; 0.09)	0.61	0.04 (-0.10; 0.17)	0.57
Total protein	0.01 (-0.06; 0.07)	0.93	0.05 (-0.08; 0.84)	0.40
**Transsulfuration related**				
Homocysteine	-0.08 (-0.14; -0.01)	0.03	-0.10 (-0.38; 0.18)	0.46
Vitamin B6	0.08 (0.02; 0.15)	0.01	0.11 (-0.03; 0.24)	0.12
Vitamin B9	0.02 (-0.05; 0.08)	0.67	0.15 (0.13; 0.42)	0.29
Vitamin B12	-0.01 (-0.07; 0.06)	0.86	0.12 (-0.17; 0.42)	0.42
Sulfate excretion	0.41 (0.34; 0.47)	< 0.001	0.59 (0.47; 0.70)	< 0.001
Taurine excretion	0.15 (0.08; 0.23)	< 0.001	0.14 (0.01; 0.29)	0.048
**Other**				
Hemoglobin	0.10 (0.03; 0.17)	0.007	0.01 (-0.16; 0.17)	0.98
Hs-CRP	-0.13 (-0.21; -0.07)	< 0.001	-0.05 (-0.16; -0.07)	0.45
NT-proBNP	-0.21 (-0.28; -0.13)	< 0.001	-0.12 (-0.24; -0.01)	0.04
Glucose	-0.10 (-0.17; -0.04)	0.003	0.01 (-0.12; 0.14)	0.90
SQUASH score	0.13 (0.05; 0.20)	< 0.001	n/a	n/a
Protein intake	0.49 (0.42; 0.56)	< 0.001	0.22 (0.06; 0.39)	0.007
Calcineurin inhibitor usage	-0.07 (-0.22; 0.08)	0.37	n/a	n/a
Proliferation inhibitor usage	0.19 (0.01; 0.38)	0.05	n/a	n/a
Prednisolon dosage	-0.02 (-0.09; 0.06)	0.7	n/a	n/a

Analyses are adjusted for age, sex, weight, height and time since transplantation (for KTR)

### Associations of measured GFR with creatine parameters

Measured GFR was 57 ± 20 ml/min and 114 ± 24 ml/min in the 157 KTR and 167 controls with measured GFR filtration data, respectively.

In KTR, a higher measured GFR was associated with a higher endogenous creatine synthesis rate and higher total creatine pool. A higher measured GFR was also associated with higher plasma concentrations of both guanidinoacetate and creatine in KTR. Furthermore, a higher measured GFR was associated with higher urinary excretions of both guanidinoacetate and creatine in KTR. Lastly, a higher measured GFR was associated with a higher fractional excretion of guanidinoacetate and a lower fractional excretion of creatine in KTR. In controls, many of these associations were not found. Exceptions are the association of a higher measured GFR with higher plasma creatine concentration and higher urinary guanidinoacetate excretion. An overview of linear regression analyses of measured GFR with creatine related parameters in KTR and donors is shown in Table 4.

**Table 4:** Associations of measured GFR with creatine parameters in KTR and controls

	KTR *n* = 157 mean measured GFR: 57 ± 20 95 percentile range: 25–87		Controls *n* = 167 mean measured GFR: 114 ± 24 95 percentile range: 80–157	
Independent variable:	Std. Beta (95% CI)	*P*-value	Std. Beta (95% CI)	*P*-value
**Creatine status**				
Endogenous creatine synthesis rate	0.21 (0.08; 0.33)	0.002	0.09 (-0.09; 0.26)	0.34
Total creatine pool	0.22 (0.11; 0.33)	< 0.001	0.10 (-0.06; 0.24)	0.22
Creatine intake	0.10 (-0.05; 0.25)	0.20	0.15 (-0.05; 0.34)	0.14
**Plasma concentrations**				
Arginine	-0.13 (-0.29; 0.03)	0.09	0.15 (-0.06; 0.36)	0.16
Glycine	-0.20 (-0.35; -0.05)	0.01	-0.13 (-0.33; 0.07)	0.19
Guanidinoacetate	0.12 (-0.03; 0.28)	0.11	-0.19 (-0.47; 0.08)	0.17
Creatine	0.22 (0.08; 0.36)	0.003	0.25 (0.06; 0.44)	0.009
**Urinary excretions**				
Arginine	0.31 (0.15; 0.46)	< 0.001	0.20 (-0.01; 0.40)	0.05
Glycine	0.19 (0.04; 0.35)	0.02	0.23 (0.03; 0.43)	0.02
Guanidinoacetate	0.69 (0.57; 0.81)	< 0.001	0.47 (0.28; 0.66)	< 0.001
Creatine	0.26 (0.10; 0.41)	< 0.001	0.24 (0.04; 0.44)	0.02
**Fractional excretion**				
Arginine	-0.17 (-0.33; -0.01)	0.04	-0.26 (-0.46; -0.05)	0.01
Glycine	-0.19 (-0.34; -0.03)	0.02	0.03 (-0.18; 0.25)	0.76
Guanidinoacetate	0.32 (0.16; 0.47)	< 0.001	0.17 (-0.09; 0.43)	0.20
Creatine	-0.29 (-0.45; -0.14)	< 0.001	-0.01 (-0.22; 0.20)	0.92

Analyses are adjusted for age, sex, weight, height and time since transplantation (for KTR)

### Mediation analyses

**Fig. 4:**
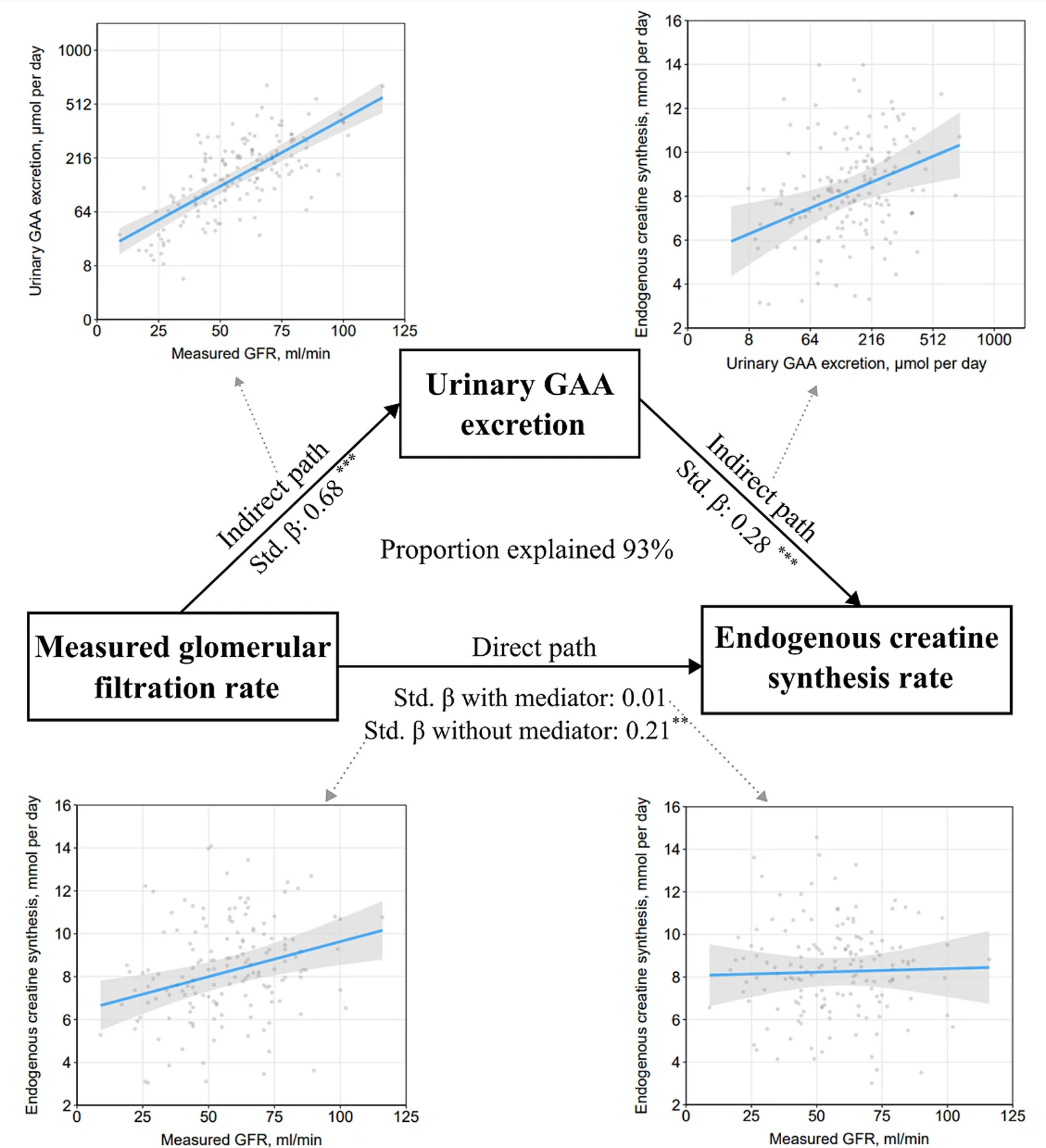
Visual representation of the mediation analyses. Prior to the mediation analyses, a strong association between measured GFR and the endogenous creatine synthesis rate was found (left bottom figure; Std. beta: 0.21). Mediation analyses demonstrated that when accounting for the mediator urinary guanidinoacetate excretion (as a marker for guanidinoacetate production), the direct path lost significance, whereas the indirect path was strongly significant. Mediation analyses demonstrated that the proportion explained was 93%. The individual plots were plotted using the visreg package, and the dots represent individual data points, with each dot indicating the predicted value of the dependent variable for a specific observation while holding other independent variables constant, as determined by the linear regression analysis. The blue line indicates the coefficient and the shaded grey area represents the 95% confidence interval. Analyses are adjusted for age, sex, weight, height and time since transplantation. ^**^and ^***^ indicate a *P*-value below 0.01 and 0.001, respectively

Mediation analyses were conducted to assess whether the association of measured GFR with the endogenous creatine synthesis (std. beta: 0.21 (95% CI: 0.08; 0.33); *P* = 0.002), could be mediated by urinary guanidinoacetate excretion (as a marker for guanidinoacetate production). In KTR, the direct path lost significance (std. beta: 0.01 (95% CI: -0.15; 0.18); *P* = 0.88), whereas the indirect path was highly significant (std. beta: 0.19 (95% CI: 0.08; 0.31); *P* < 0.001). The proportion mediated was 93% (*P* < 0.001) in KTR. In controls, there was no significant association of measured GFR with the endogenous creatine synthesis (std. beta: 0.09 (95% CI: -0.09; 0.26); *P* = 0.34), and consequently there was also no significant mediation by guanidinoacetate excretion. A tabulated and a graphical overview of the mediation analyses is shown in Table 5; Fig. 4, respectively. In sensitivity analyses, similar mediation analyses were performed on the association of measured GFR with total creatine pool, with again urinary guanidinoacetate excretion (as a marker for guanidinoacetate production) as a potential mediator. A tabulated overview of these analyses is shown in Table S6, which demonstrated that the proportion mediated by urinary guanidinoacetate excretion was 95% (*P* < 0.001) in KTR, with no mediation in controls.

**Table 5:** Mediation analyses on the association of measured GFR and endogenous creatine synthesis rate with urinary guanidinoacetate excretion as the mediator

		KTR *n* = 157 mean measured GFR: 57 ± 20 95 percentile range: 25–87		Controls *n* = 167 mean measured GFR: 114 ± 24 95 percentile range: 80–157	
**Endogenous creatine synthesis**					
	**Direct path ** **(**measured GFR -> Creatine synthesis**)**	0.01 (-0.15; 0.18)	0.88	-0.08 (-0.37; 0.21)	0.58
	**Indirect path** (measured GFR -> GAA excretion -> Creatine synthesis)	0.19 (0.08; 0.31)	< 0.001	0.21 (0.08; 0.38)	< 0.001
	**Total path** (sum of direct and indirect)	0.20 (0.08; 0.33)	< 0.001	0.14 (-0.17; 0.44)	0.35
	**Proportion mediated** (multiply by 100 for %)	0.93 (0.36; 2.55)	< 0.001	No mediation	n/a

Analyses are adjusted for age, sex, weight, height and time since transplantation (for KTR)

Abbreviations: GAA: Guanidinoacetate; GFR: Glomerular filtration rate

## Discussion

The cardinal finding of our study is that, although KTR had a similar body weight, height and dietary creatine intake as controls, the respective values of endogenous creatine synthesis rate and total creatine pool were on average 22% and 14% lower in KTR as compared to controls. Despite lower GFR, the respective concentrations of plasma guanidinoacetate and creatine concentrations were 21% and 41% lower in KTR as compared to controls. Urinary excretion of guanidinoacetate and creatine were 66% and 59% lower in KTR as compared to controls. Furthermore, we demonstrated that while arginine, glycine and creatine are actively reabsorbed in the kidneys, guanidinoacetate was reabsorbed less extensively, approaching a fractional excretion of 100%. In KTR, higher physical activity, protein intake, vitamin B6 status, and hemoglobin were each associated with a higher endogenous creatine synthesis rate. Furthermore, lower homocysteine, hs-CRP, NT-proBNP and glucose were also associated with higher endogenous creatine synthesis rate. No association was found with biochemical liver parameters. Most associations had a similar direction in controls, but did not reach statistical significance.

In KTR, a higher measured GFR was strongly associated with a higher endogenous creatine synthesis rate as well as higher total creatine pools. Mediation analyses demonstrated that these associations were fully mediated by the urinary guanidinoacetate excretion, which may serve as a marker of guanidinoacetate production. Overall, our results indicate that KTR differ strongly from controls in terms of creatine homeostasis, with lower total creatine pools, a lower endogenous creatine synthesis rate and lower circulating concentrations of creatine. The positive relationship of kidney function with endogenous creatine synthesis rate highlights a potential role of creatine supplementation in KTR, especially for those with the most impaired kidney function. Similarly, other patients with reduced enzymatic kidney activity, most notably patients with end-stage kidney disease relying on dialysis treatment, may also potentially benefit from creatine supplementation. Currently, a pilot trial is underway investigating the effects of intradialytic creatine supplementation in hemodialysis patients (van der Veen et al. 2021).

Although ATP represents the universal energy currency in all organisms and cells, ATP levels are not easily up-regulated in cells with high and/or intermittently fluctuating energy demand. As ATP is broken down into ADP and Pi, protons (H^+^) are released, which would acidify the cytosol and inhibit ATPases essential to life. Phosphagens, which are metabolically inert high-energy compounds, circumvent this problem by providing a temporal and spatial energy buffer (Wallimann et al. 2011). In humans and vertebrate animals, creatine is the primary phosphagen. Creatine can be phosphorylated to phosphocreatine by creatine kinase to capture immediately available cellular energy, storing up to 10 times the amount in the ATP pool. Alternatively, when and where needed, phosphocreatine can release its free energy by donating the phosphate group to ADP, regenerating ATP. The ability to replenish depleted ATP levels during high-energy demand states like intense exercise or in conditions where energy production is either impaired (e.g., ischemia, hypoxia) or insufficient due to increased demand (e.g., fatigue) is important in maintaining ATP availability (Wallimann et al. 2011; Kreider and Stout 2021). The fact that the total creatine pool is also of vital importance for normal physiological kidney function, as well as in kidney diseases has been demonstrated using observational studies. In patients with CKD stage 3 to 5, a lower creatine pool, evidenced by a lower 24-h creatinine excretion, was associated with a higher risk of both mortality and initiating dialysis treatment (Di Micco et al. 2013). Furthermore, a recent study demonstrated that patients with advanced CKD decrease in 24-h creatinine excretion each year and that a higher loss is associated with more mortality after transitioning to end-stage kidney disease (Amin et al. 2024). Within both peritoneal and hemodialysis patients, a lower creatine pool was also associated with a higher risk of mortality (Perez et al. 2000; Post et al. 2018). Even after successful kidney transplantation, the total creatine pool remains a strong predictor of both graft failure and mortality (Oterdoom et al. 2009; van Vliet et al. 2022). It should be noted that in most of these studies, the 24-h urinary creatinine excretion was the variable of interest. This is directly proportional to the total creatine pool, as on a constant and daily basis a total of 1.7% of the total creatine pool is degraded to creatinine and excreted renally (Crim et al. 1975; Walker 1979). Since 95% of the total creatine pool resides in skeletal muscle mass, it is usually postulated that such associations are reflecting relations with muscle mass, rather than with creatine pools themselves. Interestingly, the association of total creatine pool with mortality remained significant after adjusting for muscle mass (measured via bioimpedance analysis) or muscle strength (van Vliet et al. 2022). Furthermore, in a recent study, we demonstrated that the total creatine pool indexed to muscle mass is associated with self-reported fatigue in KTR, indicating that the creatine pool is clinically relevant, even when corrected for absolute muscle mass (Post et al. 2023).

In the current study we demonstrated that KTR had a similar body weight and height, as well as a comparable dietary creatine intake compared with controls. Despite this, the total creatine pool was on average 14% lower, and the endogenous creatine synthesis was even 22% lower than controls. Similar trends were found for the plasma concentration and urinary excretion of guanidinoacetate and creatine. Plasma guanidinoacetate and plasma creatine concentrations were 21% and 41% lower in KTR as compared to controls, respectively. Urinary excretions of guanidinoacetate and creatine were 66% and 59% lower in KTR as compared to controls. The lower endogenous creatine synthesis is likely caused by a reduced AGAT activity/expression in the kidneys, which catalyzes the first and rate-limiting step of creatine synthesis, the production of guanidinoacetate from arginine and glycine. It is less likely that substrate deficiency is the cause of reduced endogenous creatine synthesis, as the concentrations of arginine and glycine in plasma were both higher in KTR as compared to controls.

Besides being involved in the endogenous synthesis of creatine, some kidney epithelial cells express high levels of CK, especially those in the proximal tubule that are highly metabolically active due to large transcellular ion fluxes driven by ion-pump ATPases, (14). Similar to muscle and brain cells, they depend on energy-rich phosphocreatine for temporal and spatial energy buffering. Thus, efficient proximal tubular epithelial cell function requires an optimal supply of creatine as a precursor for phosphocreatine (Li et al. 2010). In line with previous research, we found that the amino acids arginine and glycine are heavily reabsorbed, with fractional excretions below 1%, indicating > 99% reabsorption (Makrides et al. 2014). Creatine and guanidinoacetate are both small molecules filtered in the glomeruli and partially reabsorbed by the Na^+^ and Cl^−^ -dependent creatine transporter SLC6A8 (also known as CRT1) in the apical membrane of the proximal tubular cells. After reabsorption into the tubular cells, creatine is transported into the circulation via the monocarboxylate transporter, SLC16A12, localized in the basolateral membrane (Verouti et al. 2021). While the trans-cellular pathway for creatine is known, the basolateral exit pathway of guanidinoacetate, which is both reclaimed and synthesized by proximal tubular cells, remains currently unknown (Verouti et al. 2021). The first study on renal handling of creatine found a nearly complete reabsorption of creatine, with no urinary creatine detected (Sims and Seldin 1949). In a more recent study, creatine reabsorption rates were in the range of 90–100%, which is in line with the findings in our study, with a fractional excretion of 4% for the control group and 6% fractional excretion in the KTR group (Dhayat et al. 2016). Guanidinoacetate, although also a substrate for the SLC6A8 transporter, is reabsorbed much less extensively. Two clinical studies demonstrated that the fractional of guanidinoacetate varies roughly between 75 and 100%, which is in line with the findings in our control group, where the fractional excretion was 80%, while it was only 71% in KTR (Dhayat et al. 2016; Mikuteit et al. 2023). It should be noted, however, that fractional excretion refers to the net process and could potentially be a combination of both secretion and reabsorption. It has previously been hypothesized that some of the renally synthesized guanidinoacetate is lost into the urine through unidentified mechanisms (Kiyatake et al. 2004). Indeed, in our study one third of patients had a fractional excretion of guanidinoacetate larger than 100%, indicating net secretion. Furthermore, a study on a Swiss family with a heterozygous SLC16A12 mutation reported fractional guanidinoacetate excretions by far exceeding 100% in several members, while those of controls were < 100%. It has been hypothesized that in the absence of SLC16A12, the intracellular concentrations of creatine will rise, which in turns inhibit SLC6A8 transporter activity, inhibiting both creatine and guanidinoacetate reabsorption (Dhayat et al. 2016; Verouti et al. 2021). Although much remains to be elucidated regarding the renal handling of creatine and guanidinoacetate, the findings of both KTR and control were generally in line with those in previous studies.

In our linear regression analyses, of all the creatine related variables, the urinary guanidinoacetate excretion was most strongly associated with the endogenous creatine synthesis rate, both in KTR and controls. We hypothesized that urinary guanidinoacetate reflects the guanidinoacetate production by organs that contain high amounts of AGAT and low to little amounts of GAMT, most notably the kidney and in lesser extent the pancreas (Van Pilsum et al. 1972; Da Silva et al. 2014). Since the first step of the creatine synthesis is considered the rate limiting step, the rate of guanidinoacetate release in the circulation from these organs will drive the circulating guanidinoacetate concentration. The circulating guanidinoacetate concentrations together with the glomerular filtration rate subsequently determine the filtered load. After subsequent handling, which in the case of guanidinoacetate is net limited reabsorption given the high fractional excretion, the remainder is excreted in the urine. In steady state, alterations to the renal handling will primarily influence the plasma concentration and not the urinary excretion. For instance, an increase in tubular reabsorption will increase the plasma concentration, which in turn increases the filtered load, compensating the increased reabsorption and over time, in steady state, leading to a comparable 24-h excretion. This may explain why the endogenous creatine synthesis was more strongly associated with urinary guanidinoacetate excretion as compared to plasma guanidinoacetate. It should be noted that guanidinoacetate dietary intake may also contribute to circulating guanidinoacetate concentrations and urinary guanidinoacetate excretion, although a recent study in NHANES reported that food-driven guanidinoacetate appears to provide approximately 0.5% of the creatine homeostatic load in a regular diet, so that this contribution is likely limited (Ostojic et al. 2022).

Therefore, we hypothesize that the 24-h urinary guanidinoacetate excretion is a reflection of the total guanidinoacetate production rate in tissues that express little or no GAMT. It should also be noted that certain organs may possess both AGAT and GAMT, which has been demonstrated unequivocally for the brain (Braissant et al. 2007), which will likely not contribute to circulating guanidinoacetate or guanidinoacetate excretion.

Of interest, the associations of estimations of GFR with the endogenous creatine synthesis rate and the total creatine pool demonstrated that eGFR based on creatinine alone was not associated with endogenous creatine synthesis rate and total creatine pool, whereas the cystatin C based eGFR, creatinine clearance and measured GFR were all positively associated with both the endogenous creatine synthesis rate and total creatine pool. This discrepancy can theoretically be explained by the fact that circulating creatinine concentrations are influenced in opposite directions by the creatine pool and the GFR. The creatine pool is directly responsible for the synthesis and thus supply of creatinine to the circulation, while the GFR is a reflection of the primary route of elimination of creatinine. Therefore, to assess whether there is a relationship between GFR and the endogenous creatine synthesis rate or creatine pool, it is not appropriate to use a creatinine-based estimate of GFR. Patients with a higher creatine pool will have a higher supply of creatinine to the circulation (which lowers their estimation of eGFR if only based on creatinine), which in turn would cancel out a true underlying positive association of GFR with endogenous creatine synthesis or total creatine pools or strengthen a underlying true negative association. Therefore, in the current study, we also included muscle mass independent measures, including cystatin C-based eGFR, creatinine clearance, and most importantly, measured GFR. All these demonstrated a strong and significant association of kidney function with endogenous creatine synthesis rate and total creatine pool. This supports the notion that creatine synthesis is regulated at the level of AGAT expression, and that the kidney is an important source of AGAT activity. In line with this, no associations were found between the endogenous creatine synthesis rate and markers of liver function, including the transaminases, bilirubin and total protein levels.

It should be noted, however, that the associations of eGFR_Cys C_ / eGFR_Cys C-Creat_ with endogenous creatine synthesis rate were only found in KTR and not in the control group. It could be hypothesized that this is related to the smaller sample size, but this is unlikely as the association of measured GFR with endogenous creatine synthesis rate was also only found in KTR and not in controls (while sample sizes were similar for those analyses). Similarly, the association of measured GFR with total creatine pools was only evidenced in KTR and not in controls. A possible explanation might be that under physiological circumstances, the variation in kidney function does not impact overall endogenous creatine synthesis rate, given the potential reserve capacity of healthy kidneys, or other extrarenal sources of guanidinoacetate and creatine production, such as in the pancreas (Edison et al. 2007). However, it is possible that when the system is under stress by serious illness and impaired kidney function, the remaining kidney function becomes increasingly important for the rate-limiting production of guanidinoacetate and consequently the overall endogenous creatine synthesis rate.

Strengths of our study are the wide variety of biochemical parameters related to creatine measured in parallel, as well as the use of actual GFR measurements. However, we did not measure phosphocreatine. This is because phosphocreatine concentrations is generally believed to be found primarily in tissues and only in negligible amounts in the circulation. This is supported by a study into the pharmacokinetics of exogenous phosphocreatine, which previously demonstrated that all of the pre-dose plasma samples had phosphocreatine concentrations below the limit of quantitation of 1.96 µmol/L (He et al. 2020). Plasma phosphocreatine concentrations of the same order of magnitude were found in metastatic colorectal cancer patients (Ma et al. 2023). Furthermore, non-clinical pharmacokinetic study demonstrated that nearly three fourth of an intravenously administrated phosphocreatine dose was quickly converted into creatine (Xu et al. 2014). However, it should be acknowledged that we also did not measure creatine, nor phosphocreatine in the skeletal muscles of patients. Although this would be of great interest, the invasiveness of a biopsy procedure made it unsuitable for a large observational and biobanking study. Furthermore, it remains a matter of debate what the actual bioavailability of creatine in dietary sources is. For this study, we assumed a full bioavailability, as previous studies were unable to determine creatine and creatinine in feces (Harris et al. 2002; Deldicque et al. 2008), and the bioavailability of creatine monohydrate is believed to be 100% (Kreider et al. 2022). However, it remains an uncertainty, as there are also indications that a certain proportion of creatine can be converted to creatinine in the acidic environment of the stomach. Also, at this point it is unfortunately unknown what cut-off value of creatine pools would be able to discriminate between insufficient and sufficient creatine pools. A trial of creatine supplementation could help clarify such issues. Lastly, as stated before, the observational nature precludes statements regarding causality.

To summarize, in this study we performed an extensive comparison of creatine homeostasis of KTR to controls. While KTR had a similar body weight, height and dietary creatine intake as controls, the endogenous creatine synthesis rate and the total creatine pool were on average 22% and 14 lower in KTR as compared to controls. Furthermore, the concentrations of plasma guanidinoacetate and creatine were 21% and 41% lower in KTR as compared to controls. Urinary excretion of guanidinoacetate and creatine were 66% and 59% lower in KTR as compared to controls. Importantly, in KTR, but not controls, there was a strong direct association of measured GFR with the endogenous creatine synthesis rate and the total creatine pool, highlighting the importance of kidney function on creatine homeostasis in KTR. Based on the strong associations of urinary guanidinoacetate with both endogenous creatine synthesis rate and measured GFR, we hypothesized that urinary guanidinoacetate is a reflection of guanidinoacetate production by organs with AGAT activity but less to no GAMT activity, most notably the kidneys. We therefore aimed to test whether the positive association of measured GFR with endogenous creatine synthesis rate was mediated by the urinary guanidinoacetate excretion. These analyses indeed demonstrated that the association was fully mediated by the urinary guanidinoacetate excretion, supporting the notion that indeed the first step of creatine synthesis rate is the rate-limiting step. Similarly, the association of measured GFR with the total creatine pool was also fully mediated by urinary guanidinoacetate excretion. Taken together, our findings highlight that KTR have a disturbed creatine homeostasis as compared to controls and in those with the lower kidney function, creatine supplementation may be of use, given the direct relationship of measured GFR and endogenous creatine synthesis rate. In line with the findings documented herein, is the fact that dialysis patients also present with a disturbed creatine metabolism, with a lower total creatine pool size as compared to healthy individuals (Post et al. 2019, 2021b). Taken together, it seems likely that not only chronic dialysis patients should benefit from creatine supplementation, as proposed earlier (Wallimann et al. 2017; Post et al. 2019), but also KTR, as suggested here. However, further research is clearly needed on these important topics.

## Electronic supplementary material

Below is the link to the electronic supplementary material.

Supplementary material 1

## Data Availability

Data described in the manuscript, code book, and analytic code will be made available upon request of the editor.
