# Supplementary material for: Creatine homeostasis and the kidney: comparison between kidney transplant recipients and healthy controls
Source: Amino Acids. 2024 Jun 13;56:42. doi: 10.1007/s00726-024-03401-w (PMC11176230; doi:10.1007/s00726-024-03401-w)
Supplement: Supplementary file 1 [file 726_2024_3401_MOESM1_ESM.docx]

**Supplementary data**

**Figure S1.** Flowchart of KTR and donors in the study.

| **Table S1.** Baseline characteristics in KTR and controls | | | |
| --- | --- | --- | --- |
|  | **KTR (n=553)** | **Controls (n=168)** | **P-value** |
| **Creatine related** |  |  |  |
| Total creatine pool, mmol | 651 ± 178 | 753 ± 239 | <0.001 |
| Creatine intake, mmol/day | 3.5 ± 1.2 | 3.5 ± 1.4 | 0.7 |
| Endogenous creatine synthesis rate, mmol/day | 7.9 ± 3.0 | 10.0 ± 4.1 | <0.001 |
| **Demographics** |  |  |  |
| Age, years | 53.1 ± 12.9 | 53.9 ± 10.7 | 0.4 |
| Sex, n male (%) | 316 (57%) | 79 (47%) | 0.03 |
| Weight, kg | 80.1 ± 16.4 | 80.2 ± 13.8 | 0.9 |
| Height, cm | 174 ± 10 | 175 ± 9 | 0.1 |
| **Kidney related** |  |  |  |
| eGFR creat+cys 2021, ml/min/1.73m^2^ | 47 ± 20 | 98 ± 15 | <0.001 |
| eGFR creat 2021, ml/min/1.73m^2^ | 56 ± 21 | 94 ± 14 | <0.001 |
| eGFR cys C 2012, ml/min/1.73m^2^ | 42 ± 19 | 96 ± 19 | <0.001 |
| Creatinine clearance, ml/min | 63 ± 30 | 136 ± 62 | <0.001 |
| **Liver related** |  |  |  |
| ALAT, U/L | 19 [14; 25] | 22 [17; 30] | <0.001 |
| ASAT, U/L | 22 [18; 27] | 23 [21; 26] | 0.01 |
| Billirubin, µmol/L | 10 [8; 14] | 8 [6; 11] | <0.001 |
| Total protein, g/L | 71 ± 5 | 74 ± 4 | <0.001 |
| **Transsulfuration related** |  |  |  |
| Homocysteine, µmol/L | 20 [16; 25] | 11 [10; 15] | <0.001 |
| Vitamin B6, nmol/L | 30 [19; 55] | 43 [29; 65] | <0.001 |
| Vitamin B9, nmol/L | 19 [14; 27] | 22 [17; 27] | 0.03 |
| Vitamin B12, pmol/L | 288 [222; 377] | 263 [213; 354] | 0.3 |
| Sulfate excretion, mmol/24h | 17.8 ± 6.5 | 18.8 ± 6.0 | 0.07 |
| Taurine excretion, µmol/24h | 518 [217; 952] | 495 [232; 966] | 0.7 |
| **Other** |  |  |  |
| Hemoglobin, mmol/L | 8.3 ± 1.1 | 8.9 ± 0.8 | <0.001 |
| Hs-CRP, mg/L | 1.6 [0.7; 4.4] | 1.1 [0.5; 1.9] | <0.001 |
| NT-proBNP, ng/L | 254 [104; 608] | 39 [22; 68] | <0.001 |
| Glucose, mmol/L | 5.3 [4.8; 5.9] | 5.3 [5.0; 5.7] | 0.3 |
| SQUASH score, hours×intensity | 5160 [2520; 7980] | n/a | n/a |
| Protein intake, g/day | 84 ± 21 | 87 ± 20 | 0.07 |
| Calcineurin inhibitor usage, n (%) | 311 (56%) | n/a | n/a |
| Proliferation inhibitor usage, n (%) | 459 (83%) | n/a | n/a |
| Prednisolon dosage, mg/24h | 10 [5; 10] | n/a | n/a |
| Differences between baseline characteristics in KTR and controls were tested using the student t-test, Wilcoxon signed-rank test and Chi-squared test, where appropriate. | | | |

| **Table S2.** Sensitivity analyses of a comparison of creatine pool and muscle mass in selected data from the TransplantLines cohort. | | | |
| --- | --- | --- | --- |
|  | **KTR (n=397)** | **Controls (n=428)** | **P-value** |
| **Muscle mass** |  |  |  |
| Appendicular muscle mass (Sergi), kg | 20.6 ± 4.3 | 21.1 ± 4.8 | 0.1 |
| Appendicular muscle mass (Kyle), kg | 22.0 ± 5.0 | 22.3 ± 5.4 | 0.3 |
| Total muscle mass (Janssen), kg | 26.4 ± 6.6 | 25.6 ± 6.9 | 0.1 |
| **Creatine pool** |  |  |  |
| Creatine pool, mmol | 695 ± 208 | 759 ± 246 | <0.001 |
| **Ratio creatine pool to muscle mass** |  |  |  |
| Ratio based on appendicular muscle mass (Sergi), mmol/kg | 33.9 ± 7.7 | 35.9 ± 7.8 | <0.001 |
| Ratio based on appendicular muscle mass (Kyle), mmol/kg | 31.9 ± 7.3 | 34.0 ± 7.3 | <0.001 |
| Ratio based on total muscle mass (Janssen), mmol/kg | 26.7 ± 6.3 | 29.9 ± 6.5 | <0.001 |
| Differences between baseline characteristics in KTR and controls were tested using the student t-test. | | | |

| **Table S3.** Comparison of fractional excretions of creatine and its precursors in and controls in the mGFR dataset (part 2 analysis). | | | |
| --- | --- | --- | --- |
|  | **KTR (n=157)** | **Controls (n=167)** | **P-value** |
| **Fractional renal excretion**  **(based on creatinine clearance)** |  |  |  |
| Arginine, % | 0.32 [0.21; 0.46] | 0.18 [0.13; 0.24] | <0.001 |
| Glycine, % | 4.5 [2.8; 7.6] | 4.5 [3.1; 5.9] | 0.7 |
| Guanidinoacetate, % | 69 [45; 105] | 80 [55; 112] | 0.1 |
| Creatine, % | 5.0 [2.9; 8.0] | 4.0 [2.5; 8.7] | 0.1 |
| **Fractional renal excretion**  **(based on measured GFR)** |  |  |  |
| Arginine, % | 0.33 [0.23; 0.48] | 0.19 [0.14; 0.27] | <0.001 |
| Glycine, % | 4.7 [3.1; 8.0] | 4.9 [3.5; 6.7] | 0.8 |
| Guanidinoacetate, % | 79 [53; 112] | 83 [63; 114] | 0.2 |
| Creatine, % | 5.4 [3.5; 8.7] | 4.7 [2.9; 10.2] | 0.1 |
|  | | | |

| **Table S4.** Associations of creatine parameters with the total creatine pool in KTR and controls | | | | |
| --- | --- | --- | --- | --- |
|  | **KTR**  (n=553) | | **Controls**  (n=168) | |
|  | **Std. Beta (95% CI)** | **P-value** | **Std. Beta (95% CI)** | **P-value** |
| **Creatine status** |  |  |  |  |
| Endogenous creatine synthesis rate | 0.77 (0.73; 0.81) | <0.001 | 0.64 (0.57; 0.71) | <0.001 |
| Creatine intake | 0.11 (0.05; 0.17) | <0.001 | 0.07 (-0.04; 0.17) | 0.20 |
| **Plasma concentrations** |  |  |  |  |
| Arginine | -0.01 (-0.07; 0.06) | 0.89 | -0.01 (-0.10; 0.09) | 0.90 |
| Glycine | -0.02 (-0.07; 0.05) | 0.62 | 0.02 (-0.09; 0.12) | 0.76 |
| Guanidinoacetate | 0.11 (0.05; 0.17) | <0.001 | 0.05 (-0.06; 0.15) | 0.39 |
| Creatine | 0.06 (-0.01; 0.13) | 0.06 | -0.01 (-0.11; 0.10) | 0.93 |
| **Urinary excretions** |  |  |  |  |
| Arginine | 0.15 (0.09; 0.21) | <0.001 | 0.14 (0.03; 0.24) | 0.007 |
| Glycine | 0.20 (0.14; 0.26) | <0.001 | 0.10 (-0.01; 0.20) | 0.06 |
| Guanidinoacetate | 0.30 (0.24; 0.35) | <0.001 | 0.22 (0.13; 0.32) | <0.001 |
| Creatine | 0.22 (0.16; 0.28) | <0.001 | 0.10 (0.01; 0.20) | 0.05 |
| **Fractional excretion** |  |  |  |  |
| Arginine | -0.08 (-0.14; -0.02) | 0.009 | 0.01 (-0.10; 0.10) | 0.97 |
| Glycine | 0.01 (-0.06; 0.07) | 0.84 | -0.02 (-0.12; 0.08) | 0.67 |
| Guanidinoacetate | 0.13 (0.07; 0.19) | <0.001 | 0.02 (-0.08; 0.12) | 0.68 |
| Creatine | 0.03 (-0.03; 0.09) | 0.36 | 0.05 (-0.05; 0.15) | 0.30 |
| Analyses are adjusted for age, sex, weight, height and time since transplantation (for KTR). | | | | |

| **Table S5.** Associations of creatine parameters with the total creatine pool in KTR and controls | | | | |
| --- | --- | --- | --- | --- |
|  | **KTR**  (n=553) | | **Controls**  (n=168) | |
|  | **Std. Beta (95% CI)** | **P-value** | **Std. Beta (95% CI)** | **P-value** |
| **Demographics** |  |  |  |  |
| Age | -0.20 (-0.26; -0.14) | <0.001 | -0.29 (-0.39; -0.19) | <0.001 |
| Sex, male | 0.66 (0.50; 0.82) | <0.001 | 0.73 (0.46; 0.99) | <0.001 |
| Weight | 0.34 (0.27; 0.42) | <0.001 | 0.47 (0.34; 0.60) | <0.001 |
| Height | 0.15 (0.06; 0.24) | <0.001 | 0.01 (-0.15; 0.16) | 0.98 |
| **Kidney function markers** |  |  |  |  |
| eGFR creat+cys 2021 | 0.11 (0.05; 0.17) | <0.001 | -0.01 (-0.12; 0.10) | 0.88 |
| eGFR creat 2021 | 0.03 (-0.04; 0.09) | 0.42 | -0.06 (-0.18; 0.05) | 0.30 |
| eGFR cys C 2012 | 0.16 (0.10; 0.22) | <0.001 | 0.03 (-0.09; 0.14) | 0.64 |
| Creatinine clearance | 0.36 (0.31; 0.42) | <0.001 | 0.21 (0.11; 0.31) | <0.001 |
| **Liver function markers** |  |  |  |  |
| ALAT | 0.02 (-0.04; 0.08) | 0.53 | 0.01 (-0.10; 0.10) | 0.98 |
| ASAT | 0.03 (-0.03; 0.09) | 0.31 | 0.03 (-0.07; 0.13) | 0.58 |
| Billirubin | 0.05 (-0.01; 0.11) | 0.10 | 0.01 (-0.10; 0.10) | 0.99 |
| Total protein | 0.02 (-0.04; 0.08) | 0.51 | -0.01 (-0.10; 0.10) | 0.99 |
| **Transsulfuration related** |  |  |  |  |
| Homocysteine | -0.10 (-0.16; -0.04) | 0.001 | 0.03 (-0.14; 0.20) | 0.68 |
| Vitamin B6 | 0.09 (0.03; 0.15) | 0.003 | 0.11 (0.01; 0.21) | 0.03 |
| Vitamin B9 | 0.03 (-0.04; 0.08) | 0.69 | -0.01 (-0.17; 0.17) | 0.98 |
| Vitamin B12 | 0.02 (-0.04; 0.08) | 0.53 | 0.11 (-0.06; 0.29) | 0.21 |
| Sulfate excretion | 0.43 (0.37; 0.48) | <0.001 | 0.41 (0.32; 0.50) | <0.0001 |
| Taurine excretion | 0.20 (0.14; 0.26) | <0.001 | 0.15 (0.05; 0.25) | 0.005 |
| **Other** |  |  |  |  |
| Hemoglobin | 0.11 (0.05; 0.17) | <0.001 | -0.02 (-0.14; 0.10) | 0.76 |
| Hs-CRP | -0.11 (-0.17; -0.05) | <0.001 | -0.03 (-0.13; 0.07) | 0.58 |
| NT-proBNP | -0.19 (-0.26; -0.13) | <0.001 | -0.07 (-0.17; 0.03) | 0.18 |
| Glucose | -0.09 (-0.15; -0.03) | 0.004 | 0.02 (-0.08; 0.12) | 0.70 |
| SQUASH score | 0.15 (0.09; 0.21) | <0.001 | n/a | n/a |
| Protein intake | 0.52 (0.46; 0.58) | <0.001 | 0.40 (0.30; 0.49) | <0.001 |
| Calcineurin inhibitor usage | -0.04 (-0.18; 0.09) | 0.6 | n/a | n/a |
| Proliferation inhibitor usage | 0.17 (0.00; 0.32) | 0.05 | n/a | n/a |
| Prednisolon dosage | -0.01 (-0.08; 0.05) | 0.7 | n/a | n/a |
| Analyses are adjusted for age, sex, weight, height and time since transplantation (for KTR). | | | | |

| **Table S6.** Mediation analyses on the association of measured GFR and total creatine pool with urinary guanidinoacetate excretion as the mediator. | | | | | |
| --- | --- | --- | --- | --- | --- |
|  | | **KTR**  n= 157  mean measured GFR: 57 ± 20  95 percentile range: 25 – 87 | | **Controls**  n= 167  mean measured GFR: 114 ± 24  95 percentile range: 80 – 157 | |
| **Total creatine pool** | |  |  |  |  |
|  | **Direct path  (**measured GFR -> Creatine pool**)** | 0.02 (-0.12;0.15) | 0.81 | -0.01 (-0.16; 0.14) | 0.86 |
|  | **Indirect path**  (measured GFR -> GAA excretion -> Creatine pool) | 0.21 (0.12; 0.32) | <0.001 | 0.10 (0.04; 0.18) | <0.001 |
|  | **Total path**  (sum of direct and indirect) | 0.22 (0.10; 0.33) | <0.001 | 0.09 (-0.06; 0.24) | 0.23 |
|  | **Proportion mediated** (multiply by 100 for %) | 0.95 (0.46; 2.09) | <0.001 | No mediation | n/a |
| Analyses are adjusted for age, sex, weight, height and time since transplantation (for KTR).  Abbreviations: GAA: Guanidinoacetate; GFR: Glomerular filtration rate. | | | | | |
